# Evaluating clinical pharmacists’ involvement in postoperative acute pain services: a multicenter survey in Guangdong Province, China

**DOI:** 10.3389/fphar.2023.1283071

**Published:** 2023-10-02

**Authors:** Junxiong Lu, Mingzhen Chen, Guansheng He, Binwei Chen, Ruolun Wang

**Affiliations:** ^1^ Department of Pharmacy, The Second Affiliated Hospital of Guangzhou Medical University, Guangzhou, Guangdong, China; ^2^ Department of Orthopedics, The Second Affiliated Hospital of Guangzhou Medical University, Guangzhou, Guangdong, China

**Keywords:** clinical pharmacists, postoperative acute pain services, multicenter survey, questionnaire survey, surgical pharmacy

## Abstract

**Objective:** Postoperative pain management is an important part of surgical pharmacy. Postoperative acute pain services in China are in their initial stages. This survey aimed to investigate the attitudes, involvement, and knowledge of clinical pharmacists in China regarding postoperative acute pain services. The results can provide valuable information to guide clinical pharmacists in developing targeted strategies to improve their postoperative acute pain service capabilities.

**Methods:** A questionnaire was distributed to the pharmacy departments of 133 grade A tertiary hospitals in Guangdong province, and the responses were collected electronically.

**Results**: 123 completed questionnaires were collected from clinical pharmacists. Although 95.93% of clinical pharmacists believed they should participate in postoperative pain services, only 62.6% reported substantial involvement. Overall satisfaction with the postoperative pain service was 93.5%. Understanding of non-steroidal anti-inflammatory drugs and opioid analgesics by clinical pharmacists was comparable (*p* > 0.05). Furthermore, 98.37% of clinical pharmacists desired systematic learning in postoperative pain management, and 40.65% expressed a strong need.

**Conclusion:** Clinical pharmacists in China demonstrate a positive attitude toward participating in postoperative acute pain services. However, the actual level of involvement was concerning, and the lack of systematic training and well-established work protocols may be contributing factors. Efforts should be made to establish comprehensive and standardized processes and work protocols for postoperative acute pain services and provide systematic and hierarchical professional training to enhance clinical pharmacists’ capabilities in postoperative acute pain services.

## Introduction

Postoperative pain management is one of the key members of surgical pharmacy (Zheng et al., 2020a; Wang et al., 2023; Zheng et al., 2023). Postoperative pain is the acute pain that occurs immediately after surgery. More than 80% of postoperative patients experience acute pain, and more than 75% report moderate to severe pain (Chou et al., 2016). Another study found that up to 59% of postoperative patients reported moderate or severe pain 2 weeks after discharge (Buvanendran et al., 2015). Insufficient control of postoperative pain causes extreme suffering and more postoperative complications but can also lead to chronic pain and significantly affect patient quality of life (Kent et al., 2017). The acute pain service (APS) team (Deni, et al., 2019) and interdisciplinary collaboration (Rockett et al., 2017) are potentially effective approaches to treating postoperative pain. Pharmacists can play a crucial role in multidisciplinary collaboration by participating in APS (Zheng et al., 2020b; Janbakhsh et al., 2022). However, surveys conducted in the United States and Europe show that despite some progress in managing postoperative acute pain, more than half of patients are unsatisfied with their pain control (Gan et al., 2014; Tawfic et al., 2017). One of the reasons for this suboptimal pain management is the low level of expertise among members of the APS team (Erlenwein et al., 2016; Tawfic et al., 2017). A survey conducted in the Netherlands found that only 8% of APS teams had consultants with specialized pain education. A study on acute pain services in Canadian teaching hospitals revealed that only 11.11% of APS teams included pharmacists, and only a small fraction of them had received professional pain education (van Boekel et al., 2015; Tawfic, et al., 2021).

In China, the pain treatment survey focuses mainly on “cancer pain.” Most respondents are physicians and nurses, and there are very few investigations involving clinical pharmacists (Lian et al., 2011; Wu et al., 2023). Medical institutions in China started to provide postoperative acute pain services relatively late, and there is only a small number of clinical pharmacists (surgical pharmacists) specializing in postoperative acute pain services. Clinical pharmaceutical care services for acute postoperative pain are still in the initial stages in China (Liu et al., 2017). In particular, there is currently no large-scale survey on the status of acute pain services of clinical pharmacists in China, resulting in a lack of information on the actual capabilities of clinical pharmacists to provide postoperative APS. This study explored attitudes, behaviors, and professional knowledge about acute postoperative pain management among clinical pharmacists in tertiary grade A hospitals (highest level) in Guangdong province through a questionnaire survey. The information collected can guide surgical pharmacists to develop targeted strategies to provide postoperative APS.

## Materials and methods

### The setting of the survey and participants

The survey was conducted in Guangdong province, China. Guangdong province spans 178,000 square kilometers, approximately matching the combined land area of Greece and the Netherlands. At the end of 2022, the province had a population of 126.568 million permanent residents. Remarkably, its gross domestic product (GDP) stood at 19.9 trillion United States dollars, just shy of Italy’s GDP, which reached 20.1 trillion United States dollars within the same period. In particular, Guangdong province occupies the position of China’s premier economy and is in the third rank regarding medical standards (Guangdong Provincial Bureau of Statistics and National Bureau of Statistics Guangdong Survey Team, 2022).

The survey was conducted between January and December 2022 using an electronic questionnaire (Questionnaire Star APP). The Guangdong Pharmaceutical Association sent invitation letters and survey questionnaires to pharmacy departments of 133 member institutions (medical facilities) in Guangdong province, all of which have a tertiary grade-A qualification. Each medical institution was asked to have a clinical pharmacist involved in managing perioperative pain medications to complete the survey questionnaire. Inclusion criteria were clinical pharmacists who 1) hold a valid hospital pharmacist qualification certificate, 2) provide pharmaceutical care services related to pain medication management, and 3) volunteer to participate in survey research. Those who did not respond to all survey questions were ineligible and excluded from participation. The study was approved by the Ethics Committee of the Second Hospital of Guangzhou Medical University (approval date 12 November 2021, approval number 2021-ks-21).

### The questionnaire

The research was carried out by a survey team of clinical pharmacists, pain specialists, and surgeons. The team formulated the questionnaire through the following methodologies: 1) an extensive review of the pertinent literature, including guidelines and expert consensus on perioperative pain medication management and pharmaceutical care services; 2) a preliminary survey conducted among clinical pharmacists within their respective hospitals, focusing on pain medication management, followed by a comprehensive analysis of the survey findings; and 3) building on the insights gained from the previous steps, the team engaged in internal research discussions, culminating in the creation of the “Special Survey Questionnaire for Surgical Pharmacy Analgesia.”

The questionnaire has 11 questions and is structured into four sections. Part 1 (3 questions) collects general information, including practice location, education, and professional title. Part 2 (2 questions) explores clinical pharmacists’ attitudes on managing postoperative acute pain. Part 3 (3 questions) evaluates the degree of involvement of clinical pharmacists in postoperative acute pain management. Part 4 (3 questions) examines pharmacist knowledge about postoperative acute pain medications.

### Statistical analysis

Statistics were performed using SPSS 25.0 software. Categorical data are presented with numbers and percentages. χ^2^ test was used to compare the differences between the groups. P < 0.05 was considered statistically significant.

## Results

### Survey part 1: information on survey participants

The survey received responses from 123 medical institutions (clinical pharmacists), resulting in 123 valid questionnaires with a response rate of 92.48%. The survey included all 21 cities at the prefecture level within Guangdong province with most responses came from Guangzhou, Shenzhen, Foshan, Zhuhai, and Dongguan. Among the 123 clinical pharmacists, 70 (56.91%) had a bachelor’s degree, 42 (34.15%) had a master’s degree, and 11 (8.94%) had a doctoral degree. Regarding professional titles, 96 individuals (78.05%) held senior titles, 25 (20.33%) held intermediate titles, and 2 (1.62%) held junior titles.

### Survey part 2: attitudes of clinical pharmacists on managing postoperative acute pain

The second segment of the survey consists of two questions. Question 1 concerns the importance of involving clinical pharmacists in postoperative acute pain services with four response levels: “very necessary,” “relatively necessary,” “necessary,” and “optional.” Question 2 addresses the degree of demand for clinical pharmacists in terms of systematic learning about acute postoperative pain management with four response levels: “very needed,” “relatively needed,” “needed,” and “optional.”

Regarding the importance of involving clinical pharmacists in postoperative acute pain services, 61 (49.59%) respondents chose very necessary, 27 (21.95%) chose relatively necessary, 30 (24.39%) chose necessary, and 5 (4.07%) selected optional. There were no significant differences in the degree of involvement between the pharmacist’s educational level (χ^2^ 5.96, *p* = 0.428) and the professional title (χ^2^ 5.097, *p* = 0.531). Details are shown in Table 1.

**TABLE 1:** Responses on the importance of involving clinical pharmacists in postoperative acute pain services.

*n* (%)	Very necessary	Relatively necessary	Necessary	Optional	*χ2*	*p*
Total	61 (49.59)	27 (21.95)	30 (24.39)	5 (4.07)		
Educational level					5.960	0.428
Doctorate	5 (45.45)	4 (36.36)	2 (18.18)	0 (0)		
Master	24 (57.14)	8 (19.05)	10 (23.81)	0 (0)		
Bachelor	32 (45.71)	15 (21.43)	18 (25.71)	5 (7.14)		
Professional title					5.097	0.531
Senior	47 (48.96)	20 (20.83)	26 (27.08)	3 (3.13)		
Intermediate	13 (52.00)	6 (24.00)	4 (16.00)	2 (8.00)		
Junior	1 (50.00)	1 (50.00)	0 (0)	0 (0)		

Regarding the degree of demand for clinical pharmacists in terms of systematic learning of acute postoperative pain management, 50 (40.65%) participants chose very needed, 31 (25.20%) chose relatively needed, 40 (32.52%) chose needed, and 2 (1.63%) selected optional. There were no significant differences in the degree of demand between the pharmacist’s educational level (χ^2^ 5.122, *p* = 0.528) and the professional title (χ^2^ 10.628, *p* = 0.099). Table 2 shows the detailed results.

**TABLE 2:** Responses on the degree of demand for clinical pharmacists in terms of systematic learning about acute postoperative pain management.

*n* (%)	Very needed	Relatively needed	Needed	Optional	*χ2*	*p*
Total	50 (40.65)	31 (25.20)	40 (32.52)	2 (1.63)		
Educational level					5.122	0.528
Doctorate	5 (45.45)	1 (9.09)	5 (45.45)	0 (0)		
Master	17 (40.48)	14 (33.33)	11 (26.19)	0 (0)		
Bachelor	28 (40)	16 (22.86)	24 (34.29)	2 (2.86)		
Professional title					10.628	0.099
Senior	38 (39.58)	24 (25.00)	33 (34.38)	1 (1.04)		
Intermediate	12 (48.00)	5 (20.00)	7 (28.00)	1 (4.00)		
Junior	0 (0)	2 (100)	0 (0)	0 (0)		

### Survey part 3: the degree of involvement of clinical pharmacists in postoperative acute pain management

The third section of the survey has three questions. Question 1 concerns the level of participation of clinical pharmacists in APS with five response levels: “fully participated,” “moderately participated,” “generally participated,” “hardly participated,” and “no participation.” The second question addresses the degree of pharmacy education on pain medications for postoperative patients with five response levels: “education for all patients (>90%),” “education for most patients (70%–90%),” “education for some patients (20%–50%),” “education for a few patients (<20%),” and “no education.” Question 3 asks about the satisfaction of clinical pharmacists providing postoperative APS in their medical institutions with five response levels: “very satisfied,” “quite satisfied,” “satisfied,” “dissatisfied,” and “very dissatisfied.”

Regarding the level of participation of clinical pharmacists in APS, 14 participants (11.38%) chose fully participated, 33 (26.83%) chose moderately participated, 30 (24.39%) chose generally participated, 43 (34.96%) chose hardly participated, and 3 (2.44%) selected no participation. There were no significant differences in the level of participation between the pharmacist’s educational level (χ^2^ 8.632, *p* = 0.374) and the professional title (χ^2^ 3.263, *p* = 0.917). Details are shown in Table 3.

**TABLE 3:** Responses on the degree of involvement of clinical pharmacists in postoperative acute pain services.

*n* (%)	Fully participated	Moderately participated	Generally participated	Hardly participated	No participation	*χ2*	*p*
Total	14 (11.38)	33 (26.83)	30 (24.39)	43 (34.96)	3 (2.44)		
Educational level						8.632	0.374
Doctorate	3 (27.27)	3 (27.27)	1 (9.09)	3 (27.27)	1 (9.09)		
Master	4 (9.52)	14 (33.33)	11 (26.19)	13 (30.95)	0		
Bachelor	7 (10.00)	16 (22.86)	18 (25.71)	27 (38.57)	2 (2.86)		
Professional title						3.263	0.917
Senior	12 (12.50)	27 (28.13)	24 (25.00)	31 (32.29)	2 (2.08)		
Intermediate	2 (8.00)	6 (24.00)	5 (20.00)	11 (44.00)	1 (4.00)		
Junior	0	0	1 (50.00)	1 (50.00)	0		

Regarding the degree of pharmacy education on pain medications for postoperative patients, 7 individuals (5.69%) chose education for all patients, 22 (17.89%) selected education for most patients, 61 (49.59%) chose education for some patients, 29 (13.58%) chose education for a few patients, and 4 (3.25%) selected no education. There were no significant differences in the degree of pharmacy patient education between the pharmacist educational level (χ^2^ 6.861, *p* = 0.552). However, there were significant differences in the degree of pharmacy patient education between the pharmacist’s professional title (χ^2^ 17.652, *p* = 0.024). Table 4 shows the detailed results.

**TABLE 4:** Responses on the degree of pharmacy patient education on pain medications for postoperative patients.

*n* (%)	For all patients	For most patients	For some patients	For a few patients	No education	*χ2*	*p*
Total	7 (5.69)	22 (17.89)	61 (49.59)	29 (13.58)	4 (3.25)		
Educational level						6.861	0.552
Doctorate	1 (9.09)	1 (9.09)	5 (45.45)	4 (36.36)	0		
Master	2 (4.76)	8 (19.05)	25 (59.52)	7 (16.67)	0		
Bachelor	4 (5.71)	13 (18.57)	31 (44.29)	18 (25.71)	4 (5.71)		
Professional title						17.653	*0.024*
Senior	6 (6.25)	16 (16.67)	51 (53.13)	21 (51.88)	2 (2.08)		
Intermediate	1 (4.00)	6 (24.00)	9 (36.00)	8 (32.00)	1 (4.00)		
Junior	0 (0)	0 (0)	1 (50.00)	0 (0)	1 (50.00)		

The meaning of the italic values is that their *p* < 0.05 was considered statistically significant.

Regarding the satisfaction of clinical pharmacists with postoperative APS in their medical institutions, 15 individuals (12.20%) chose very satisfied, 48 (39.02%) chose quite satisfied, 52 (42.28%) chose satisfied, 5 (4.07%) chose dissatisfied, and 3 (2.44%) selected very dissatisfied. There were no significant differences in the degree of satisfaction between the pharmacist’s educational level (χ^2^ 6.672, *p* = 0.572). However, there were significant differences in the degree of satisfaction between the pharmacist’s professional title (χ^2^ 23.405, *p* = 0.003). Table 5 shows the detailed results.

**TABLE 5:** Responses on the satisfaction of clinical pharmacists with the postoperative APS in their respective medical institutions.

*n* (%)	Very satisfied	Quite satisfied	Satisfied	Dissatisfied	Very dissatisfied	*χ2*	*p*
Total	15 (12.20)	48 (39.02)	52 (42.28)	5 (4.07)	3 (2.44)		
Educational level						6.672	0.572
Doctorate	1 (9.09)	6 (54.55)	3 (27.27)	0 (0)	1 (9.09)		
Master	4 (9.52)	19 (45.24)	17 (40.48)	1 (2.38)	1 (2.38)		
Bachelor	10 (14.29)	23 (32.86)	32 (45.71)	4 (5.71)	1 (1.43)		
Professional title						23.405	*0.003*
Senior	10 (10.42)	39 (40.63)	42 (43.75)	3 (3.13)	2 (2.08)		
Intermediate	5 (20.00)	9 (36.00)	9 (36.00)	2 (8.00)	0 (0)		
Junior	0 (0)	0 (0)	1 (50.00)	0 (0)	1 (50.00)		

The meaning of the italic values is that their *p* < 0.05 was considered statistically significant.

### Survey part 4: pharmacist knowledge of postoperative acute pain medications

The fourth section of the survey has three questions. Question 1 assesses the knowledge of preferred analgesics for postoperative patients with three choices: “non-steroidal anti-inflammatory drugs (NSAIDs),” “weak opioid analgesics,” or “potent opioid analgesics,” Question 2 asks clinical pharmacists about their proficiency in the use of NSAIDs for postoperative acute pain with four response levels: “very familiar,” “quite familiar,” “basically familiar,” and “not familiar.” Question 3 asks about the proficiency of clinical pharmacists in the use of opioid analgesics for acute postoperative pain with four response levels: “very familiar,” “quite familiar,” “basically familiar,” and “not familiar.”

Regarding the knowledge of clinical pharmacists about the preferred analgesics for postoperative patients, 104 participants (84.55%) chose NSAIDs, 14 (11.38%) chose weak opioid analgesics, and 5 (4.07%) chose potent opioid analgesics. Table 6 shows the detailed results.

**TABLE 6:** Responses on the preferred analgesics for postoperative patients.

*n* (%)	NSAIDs	Weak opioid analgesics	Strong opioid analgesics	*χ2*	*p*
Total	104 (84.55)	14 (11.38)	5 (4.07)		
Educational level				6.675	0.154
Doctorate	7 (63.64)	3 (27.27)	1 (9.09)		
Master	34 (80.95)	5 (11.90)	3 (27.27)		
Bachelor	63 (90.00)	6 (8.57)	1 (1.43)		
Professional title				2.269	0.686
Senior	79 (82.29)	12 (12.50)	5 (5.21)		
Intermediate	23 (92.00)	2 (8.00)	0 (0)		
Junior	2 (100)	0 (0)	0 (0)		

Regarding the clinical pharmacists’ proficiency in the use of NSAIDs for acute postoperative pain, 18 (14.63%) participants chose very familiar, 57 (46.34%) chose quite familiar, 46 (37.40%) chose basically familiar, and 2 (1.63%) selected not familiar. There were no significant differences in the proficiency level between the pharmacist’s educational level (χ^2^ 3.197, *p* = 0.784) and the professional title (χ^2^ 4.953, *p* = 0.550). Table 7 shows the detailed results.

**TABLE 7:** Responses on the clinical pharmacists’ proficiency in the use of NSAIDs for postoperative acute pain.

*n* (%)	Very familiar	Quite familiar	Basically familiar	Not familiar	*χ2*	*p*
Total	18 (14.63)	57 (46.34)	46 (37.40)	2 (1.63)		
Educational level					3.197	0.784
Doctorate	1 (9.09)	5 (45.45)	5 (45.45)	0 (0)		
Master	9 (21.43)	19 (45.24)	13 (30.95)	1 (2.38)		
Bachelor	8 (11.43)	33 (47.14)	28 (40.00)	1 (1.43)		
Professional title					4.953	0.550
Senior	14 (14.58)	47 (48.96)	34 (35.42)	1 (1.04)		
Intermediate	4 (16.00)	10 (40.00)	10 (40.00)	1 (4.00)		
Junior	0 (0)	0 (0)	2 (100)	0 (0)		

Regarding clinical pharmacist’s ability to use opioid analgesics for acute postoperative pain, 16 (12.01%) participants chose very familiar, 52 (42.28%) chose quite familiar, 51 (41.41%) chose basically familiar, and 4 (3.25%) selected not familiar. There were no significant differences in the proficiency level between the pharmacist’s educational level (χ^2^ 4.844, *p* = 0.564). However, there were significant differences in the proficiency level between the pharmacist’s professional title (χ^2^ 15.650, *p* = 0.016). Table 8 shows the detailed results.

**TABLE 8:** Responses on the clinical pharmacists’ proficiency in the use of opioid analgesics for postoperative acute pain.

*n* (%)	Very familiar	Quite familiar	Basically familiar	Not familiar	*χ2*	*p*
Total	16 (12.01)	52 (42.28)	51 (41.41)	4 (3.25)		
Educational level					4.844	0.564
Doctorate	3 (27.27)	3 (27.27)	5 (45.45)	0 (0)		
Master	6 (14.29)	20 (47.62)	14 (33.33)	2 (4.76)		
Bachelor	7 (10.00)	29 (41.43)	32 (45.71)	2 (2.86)		
Professional title					15.650	*0.016*
Senior	12 (12.50)	43 (44.79)	39 (40.63)	2 (2.08)		
Intermediate	4 (16.00)	9 (36.00)	11 (44.00)	1 (4.00)		
Junior	0 (0)	0 (0)	1 (50.00)	1 (50.00)		

The meaning of the italic values is that their *p* < 0.05 was considered statistically significant.

When comparing pharmacist proficiency levels in using NSAIDs and opioid analgesics for acute postoperative pain, no significant differences were found (χ^2^ 1.271, *p* = 0.736).

## Discussion

Postoperative acute pain is a common occurrence that frequently triggers panic among patients. To effectively manage acute postoperative pain, experts, both nationally and internationally, recommend the active participation of clinical pharmacists. Despite this recommendation, there is limited research on the attitudes, behaviors, and knowledge of clinical pharmacists about acute postoperative pain services, and there is a noticeable lack of relevant surveys conducted in China. This study aimed to bridge this knowledge gap by conducting a survey that received responses from 123 tertiary grade A hospitals, representing approximately 85.42% of the total hospitals in Guangdong province. A total of 123 questionnaires were collected from clinical pharmacists, all with bachelor’s degrees or higher. Furthermore, 43.09% of the respondents had a graduate degree. All participants had pharmacist qualification certificates, with 78.05% holding senior professional titles. The high educational and professional qualifications demonstrated by the pharmacists surveyed are consistent with the overall distribution and educational background of clinical pharmacists in China (Cui et al., 2018). This valuable survey sheds light on the current state of clinical pharmacist participation in managing acute postoperative pain in China, providing a basis for future research and improvements in pain management practices.

The survey findings on the attitude toward postoperative pain management among clinical pharmacists yielded encouraging results. An overwhelming majority, 95.93%, believed they should participate in postoperative pain services. In particular, almost half of the respondents (49.59%) considered it very necessary to be involved in acute postoperative pain management, highlighting their recognition of the importance of their role in this area. The survey also did not show significant differences in attitudes based on educational background or professional titles of pharmacists, indicating a proactive and unified position among clinical pharmacists. The literature has increasingly emphasized the role of clinical pharmacists in postoperative pain management (McGonigal et al., 2017; Coulson and Kral, 2020). Collaborating closely with physicians and nurses, clinical pharmacists are pivotal in developing personalized postoperative pain management plans, leading to more comfortable postoperative acute pain treatment for patients (Wang et al., 2023).

Furthermore, the survey revealed that most clinical pharmacists (98.37%) expressed interest in acquiring systematic knowledge on postoperative pain management. A substantial portion (40.65%) expressed a strong interest in such educational opportunities, implying that the available resources in this area are insufficient to meet their professional needs. Providing comprehensive and specialized training is imperative while implementing the appropriate and pertinent management systems to address this issue. This would empower clinical pharmacists to improve their knowledge of postoperative pain management within tertiary hospitals in China (Sun et al., 2022).

Clinical pharmacists play a crucial and multifaceted role in postoperative pain management, including various essential tasks, such as pain management education, pain assessment, developing analgesic plans, monitoring treatment effectiveness, and detecting adverse reactions (Xie et al., 2023). This survey revealed that 62.6% of clinical pharmacists participated relatively well in postoperative pain management (fully, moderately, and generally participated). However, 37.4% indicated lower levels of participation (hardly participated or no participation), and no significant differences were observed based on pharmacist educational background or professional titles. This suggests that the level of involvement is consistent between different groups of clinical pharmacists. Unlike the substantial percentage of clinical pharmacists (95.93%) who expressed their willingness to participate in pain management according to the attitude survey, the actual participation of clinical pharmacists was less pronounced. This variance could be attributed to certain clinical pharmacists who have not received comprehensive training in APS, resulting in uncertainty about their practical participation in postoperative pain management tasks.

When providing patient education, the most common practice was “for some patients” (49.59%). In contrast, the percentages for “all patients” and “for most patients” were comparatively lower (5.69% and 17.89%, respectively), highlighting a certain degree of limited coverage in pain management education. However, it is crucial to recognize that most clinical pharmacists remain actively involved in patient education about pain management, with only a minority (3.25%) indicating no educational efforts. This underscores its deliberate and proactive commitment to providing pharmaceutical education about pain to patients.

Based on the satisfaction survey, a significant majority (93.5%) of clinical pharmacists expressed general satisfaction with the postoperative pain management practices within their respective medical institutions. However, there were significant differences in satisfaction levels among pharmacists with different professional titles. This disparity implies that pharmacists with lower professional titles may have inadequate pharmaceutical care capabilities, leading to suboptimal education and outcomes in pain management for this group. To address this challenge and ensure the quality and equitable provision of pain management services, it becomes imperative to prioritize efforts to enhance the pharmaceutical care capabilities of pharmacists with lower professional titles. A practical approach lies in implementing standardized training programs accessible to all clinical pharmacists, regardless of their credentials (Engle et al., 2019; Tian et al., 2021; Babu et al., 2023). The Guangdong Pharmaceutical Association has taken positive steps to promote the introduction of the concept of surgical pharmacy. This initiative involves providing systematic training to surgical pharmacists and exploring the standardization and professionalization of surgical pharmacy services (Xie et al., 2023). Through extensive and specialized training, pharmacists with lower qualifications can acquire the knowledge and skills necessary to improve their postoperative pain management performance. This standardized training methodology has the potential to bridge educational and satisfaction gaps observed among clinical pharmacists with different qualifications. Ultimately, this will lead to a more uniform and elevated level of patient care in various medical institutions.

This survey evaluated the knowledge of clinical pharmacists about the medications used in postoperative pain treatment. The key findings are as follows. Regarding the preferred choice for postoperative pain management, 84.55% of pharmacists opt for NSAIDs, which are recommended by national and international guidelines (Chou et al., 2016). This result indicates that most pharmacists made well-informed decisions, demonstrating a solid grasp of the recommended treatment approach. Clinical pharmacists exhibited a commendable understanding of both NSAIDs and opioid analgesics. Only a tiny fraction of pharmacists (1.63% and 3.25%, respectively) were unfamiliar with these drug categories.

Interestingly, the survey results pointed out that junior pharmacists had a lower understanding of opioid analgesics compared to their more experienced counterparts. To address this knowledge gap, focused training programs should be implemented to improve the understanding of junior pharmacists about opioid analgesics. This targeted approach could promote a more uniform and comprehensive knowledge level among all clinical pharmacists. Clinical pharmacists’ understanding of NSAIDs and opioid analgesics was comparable, and their proficiency in applying these two types of drugs remained consistent (Chen et al., 2018; Boren et al., 2019). This indicates competence and willingness to use these medications judiciously for postoperative pain management.

This study has several limitations. First, although the survey covered all cities at the prefecture level in Guangdong province, the distribution of tertiary hospitals was not uniform. The survey population was predominantly concentrated in economically developed areas such as Guangzhou and Shenzhen, which could affect the generalizability of the findings to other regions with distinct socioeconomic and healthcare characteristics. The survey has a small sample size. A larger sample size would have produced a more robust representation of the clinical pharmacist community. Furthermore, this research was limited to Guangdong province, renowned for its high economic status and the ranking of medical care in China. Therefore, the findings may not fully reflect the national landscape of postoperative pain management practices, especially in areas with different economic and healthcare contexts.

## Conclusion

Clinical pharmacists in China exhibit a high level of enthusiasm for the management of acute postoperative pain. The lack of systematic training and the absence of well-established and standardized work protocols, both domestically and internationally, have presented challenges in implementing comprehensive pain management services. Future efforts should focus on providing specialized training and developing standardized protocols and promoting surgical pharmacy to improve clinical pharmacist participation in postoperative pain management.
